# The Anti-Inflammatory Effect of Cabbage Leaves Explained by the Influence of bol-miRNA172a on FAN Expression

**DOI:** 10.3389/fphar.2022.846830

**Published:** 2022-03-24

**Authors:** Kaja Kasarello, Iwona Köhling, Anna Kosowska, Katarzyna Pucia, Anna Lukasik, Agnieszka Cudnoch-Jedrzejewska, Leszek Paczek, Urszula Zielenkiewicz, Piotr Zielenkiewicz

**Affiliations:** ^1^ Centre for Preclinical Research, Department of Experimental and Clinical Physiology, Medical University of Warsaw, Warsaw, Poland; ^2^ Institute of Biochemistry and Biophysics, Polish Academy of Sciences, Warsaw, Poland; ^3^ Department of Histology and Embryology, Medical University of Warsaw, Warsaw, Poland; ^4^ Department of Immunology, Transplantology and Internal Diseases, Medical University of Warsaw, Warsaw, Poland; ^5^ Faculty of Biology, University of Warsaw, Warsaw, Poland

**Keywords:** xenomiRs, rheumatoid arthritis, inflammation, collagen-induced arthritis, FAN protein

## Abstract

Recently, the possibility of cross-kingdom gene expression regulation by miRNAs from other species (“xenomiRs”), specifically from plants, has acquired scientific meaning. Based on the one of oldest methods for dealing with inflammation *via* the use of cabbage leaf compresses, we investigated the effects of *Brassica oleracea* derived miR172a on the potential human target gene encoding FAN (Factor Associated with Neutral Sphingomyelinase Activation) protein. *In vitro* experiments showed a decrease in FAN protein levels in both human and mouse cells transfected with bol-miRNA172a. As the FAN protein mediates inflammatory responses, the potential of miR172a to mitigate the inflammatory process was tested in a mouse model of rheumatoid arthritis. Animal studies showed the decreased oedema of inflamed paws in mouse with rheumatoid arthritis model induced after treatment with miR172a.

## Introduction

MicroRNAs (miRNAs) are a class of naturally occurring, small noncoding RNA molecules, usually 21–23 nucleotides long, that regulate the expression of genes in animals and plants at the posttranscriptional level. miRNAs interact with specific mRNAs through complementary base pairing to influence the translation or stability of the target mRNA molecule, typically downregulating the expression of the corresponding gene. Plant and animal miRNAs differ in sequence, processing and mode of performing similar functions. In particular, processing of the miRNA duplex in plants leads to 2′-*O*-methylation at the 3′ end by the methyltransferase HEN1 (Rogers and Chen, 2013; Baranauskė et al., 2015), which results in increased stability compared to animals.

Chen et al. (2008) were the first to find circulating miRNAs in human serum. Four years later, Zhang et al. (2011) found exogenous plant mi168a in human serum and presented preliminary results showing that this plant-encoded nucleic acid sequence specifically targets a human LDLRAP1 gene. Since this time, hundreds of studies have shown the presence of xenomiRs (exogenous miRNAs) in human fluids and tissues (Weber et al., 2010). The most spectacular evidence that plant miRNAs can enter the human body *via* the gastrointestinal tract is their presence in the milk of breastfeeding mothers (Lukasik and Zielenkiewicz, 2016), which has opened a wide discussion (Benmoussa and Provost, 2019) on related dietary issues. The other obvious question concerns the potential of plant miRNAs to become new natural medicine products (Lukasik and Zielenkiewicz, 2016). This subject was recently extensively reviewed e.g., (Sanchita et al., 2018; Jia et al., 2021; Li et al., 2021; Samad et al., 2021; Winkle et al., 2021).

Crushed cabbage leaves are one of the most widely used anti-inflammatory remedies in Polish folk medicine. Cabbage, due to its specific properties, has been used in natural medicine mainly for rheumatic pain, vein and lymphatic vessel inflammation, bruises, sprains, mastitis or gastrointestinal problems. Its “spectrum” of use is, however, much wider and encompasses the treatment of both internal and external diseases (Munns, 2003; Carper and Pszczołowski, 2008; Górnicka and Morex, 2011). We decided to evaluate whether these properties can be explained by the presence of relevant microRNAs in cabbage leaf juice. For this purpose, we performed high-throughput sequencing of miRNAs isolated from mature cabbage, which showed the presence and allowed us to determine the relative quantity of over 280 miRNAs in cabbage leaves (Lukasik et al., 2013). Here, we present bioinformatics analysis, which revealed that miR172a/miR172b**,** present in considerable quantities in cabbage leaves, may interact with mRNA encoding the FAN protein (see below), which belongs to the TNF-α signaling pathway.

TNF-α is an important factor regulating the adaptive and innate immune responses as well as inflammatory processes that occur in organisms. It plays a critical role in several known diseases, including autoimmune diseases, which affect up to 8% of the world’s population. TNF-α-related diseases include rheumatoid arthritis, Lesniewski-Crohn disease, type II diabetes, inflammatory bowel disease and many others (see www.cdc.gov). TNF-α triggers proinflammatory responses, essentially through its ability to promote the expression of various proinflammatory genes [KEGG PATHWAY: TNF signaling pathway - Homo sapiens (human) (genome.jp)]. The biological effects initiated by TNF-α rely on its ability to bind to and activate TNF-R1. As a consequence, molecular complexes are formed, resulting from the recruitment of multiple adaptor proteins to the intracellular TNF-R1 receptor (Montfort et al., 2010). One of those adaptor proteins is factor associated with neutral sphingomyelinase activation, FAN, which binds to a proximal membrane of TNF-R1. The proinflammatory activity of TNF-α is thus dependent on FAN expression.

FAN protein, a product of the NSMAF gene lying on the reverse strand of human chromosome 8, modulates the expression of proinflammatory proteins induced by TNF-α, e.g., IL-6, CCL5, CCL9, CCL20 and CXCL-2. Inhibition of FAN protein expression reduces the amount of the mentioned proinflammatory proteins and reduces the recruitment of lymphocytes in secondary lymphoid organs. FAN is a necessary intermediate for TNF-α-induced disease.

Rheumatoid arthritis (RA) is a human autoimmune, inflammatory disease affecting 0.5–1% of people, mostly middle-aged, and women 2.5 times more frequently than men. RA has an unknown etiology, possibly involving genetic, environmental and/or infectious factors (Kumar et al., 2016). The immune process in RA is directed to joints, particularly bone, cartilage and synovial tissue (Bullock et al., 2019). Symptoms of inflamed joints, such as swelling, pain, and deformities, lead to progressive disability of the patient (Guo et al., 2018).

Genetic and environmental factors contribute to the loss of tolerance, leading to autoantibody formation (rheumatoid factor or anti–citrullinated protein antibody) which are often observed before the onset of symptoms in RA patients. (Firestein and McInnes, 2017). Both innate and adaptive immune responses are involved in RA pathogenesis, but chronic inflammation is responsible for bone and cartilage degeneration due to osteoclast and metalloproteinase (MMP) activation by the proinflammatory cytokines (TNF-α, IL-1, IL-6). Proinflammatory cytokine released by synovial fibroblasts and macrophages causes synovial inflammation (McInnes and Schett, 2011) and are chemoattractants for other immune cells, recruited to the inflammatory site, leading to exacerbation of inflammation (Smolen et al., 2016).

As there is no efficient treatment for RA, due to its unknown etiology, most therapies are focused on inhibiting the ongoing inflammatory process. Nonsteroid anti-inflammatory drugs (NSAIDs), corticosteroids, cytostatic drugs and other disease-modifying drugs are used for RA treatment. In particular, disease modifying anti-rheumatic drugs (DMARDs) are of interest in RA therapy, with most of them acting as IL-1/IL-6/IL-17/TNF-α inhibitors or B cell inhibitors (Hyndman, 2017; Guo et al., 2018). The importance of surgical therapies in severe cases and complementary physiotherapy must be pointed out (Bullock et al., 2019).

TNF-α is considered one of the key cytokines in the pathomechanism of RA, contributing to the production of other proinflammatory cytokines, interactions between immune cells and boosting the inflammatory processes damaging the bone and cartilage. Therefore, many treatment strategies based on biological DMARDs target TNF-α -induced pathways (Scott et al., 2010).

In this paper, we aimed to analyze the possible anti-inflammatory activity of miRNA172a. We conducted *in vitro* experiments analyzing the effect of synthetic plant miR172a mimics on the FAN protein level in mammalian cells upon induction of the inflammatory pathway. Also we performed preliminary trials in an mice with collagen-induced arthritis (CIA), animal model of RA widely used in experiments focusing on the immunopathology of RA, immunomodulatory therapies or DMARDs (Malfait et al., 2001; Bevaart et al., 2010a; Billiau and Matthys, 2011).

Here, we present the anti-inflammatory effect of miR172a and preliminary data showing the ability of miR172a to ameliorate the clinical manifestations of RA in experimental animals.

## Materials and Methods

### Sequence Analysis

The sequences of the identified conserved and novel cabbage miRNAs (11) were used to predict their potential human target genes. The *Homo sapiens* 3′UTR, 5′UTR and CDS sequences, that were downloaded from the UCSC Genome Bioinformatics Site (http://genome.ucsc.edu/) and NCBI CCDS Database (www.ncbi.nlm.nih.gov/CCDS/CcdsBrowse.cgi), respectively, served as reference target genes dataset. Bioinformatics targets prediction was performed using miRanda method (http://cbio.mskcc.org/miRNA2003/miranda.html), the search procedure of which takes into accounts sequence complementarity, interspecies conservation and thermodynamic stability of the miRNA:mRNA duplex. The prediction parameters were as follows: 1) G:U base pairing was permitted but scored lower (score +2) than canonical base pairs (score +5), 2) the alignments with gaps and non-canonical base pairs in the “seed” regions (2–8 nt at the 5′ end of the molecule) were discarded, and 3) alignments with scores over 130 and minimum free energy (MFE) of the structure less than −17 kcal/mol were selected. Generated list of putative target genes was sorted by the highest alignment score and lowest MFE of the structure. To designate potential processes involving the predicted mRNA sequences and to asses a probable anti-inflammatory effect of cabbage miRNAs on human organism, the selected targets were mapped on the UniProt database (www.uniprot.org) and annotated using the Blast2GO (http://www.blast2go.com/b2ghome) software. The obtained dataset was analyzed and the most interesting human target gene for cabbage bol-miR172 was selected.

### miRNAs

The miR172a and miR172a* sequences were synthesized on the basis of bol-miR172a from *Brassica oleracea* var. c*apitata* (Lukasik et al., 2013): 5′-AGA​AUC​UUG​AUG​AUG​CUG​CAU-3′.

The mu_miR172a and mu_miR172a* sequences were designed by changing 3 nt (underlined) inside the region corresponding to the mouse FAN mRNA sequence potential binding site to preserve complementarity: 5′-AGA​AUC​UUA​AGA​AUG​CUG​CAU-3′.

The miR172 mimics were chemically synthesized in the form of single-stranded or miR172a/miR172a* and mu_miR172a/mu_miR172a* duplexes with both 5′ RNA strands phosphorylated and 3′ ends protected by 2′-O-methylation, and HPLC purified (FutureSynthesis, Poland).

### Cell Line Cultures and Transfections

Human foreskin fibroblasts (K21; NCBI GEO: GSM1227449) were cultured in MEM/EBSS medium (HyClone) supplemented with nonessential amino acids (Gibco) and 10% inactivated FBS (Lonza) at 37°C in a 5% CO_2_ atmosphere.

Mouse embryonic fibroblasts (WT MEFs; ATCC Manassas, United States) were cultured in DMEM (Gibco) supplemented with 10% FBS (Gibco), 100 U/ml penicillin and 100 μg/ml streptomycin (Gibco) at 37°C in a 5% CO_2_ atmosphere.

Details of the transfection procedures are given in the Supplementary Material.

Fibroblasts K21 were plated on 6-well plates (2.8 × 10^5^ cells/well) and the next day transfected with the miR172a-lipofectamine RNAiMAX (Invitrogen) complex (final concentration of miRNA when added to cells was 60 pM, 1 nM or 10 nM). MISSION^®^ microRNA Mimics HMC0003 (Sigma) and Hs_NSMAF_3 (QIAGEN) were used as a negative and positive control, respectively at a final concentration of 10 nM. The cells were incubated for 48 or 72 h. Four hours before the end of the experiment, LPS (final concentration 1 μg/ml) was added to each well. Then, the cell pellet was suspended in 400 µl of PBS and stored at −20°C until analysis.

MEFs were plated on 24-well plates (1.9 × 10^4^ cells/well) and the next day, 1 nM, 10 nM or 100 nM mu_miR172a/mu_miR172a* duplex was transfected into MEFs (two wells each) using ScreenFect A-plus (ScreenFect GmbH) according to the manufacturer’s instructions. The next day, the transfection procedure was repeated, and the cells were incubated for the next 24 h. As a negative control, nonspecific dsRNA (siGENOME Nontargeting siRNA #3, Dharmacon) at a concentration of 10 nM was used. Four hours before the end of the experiment, TNF-α (1 ng/ml) was added to each well. Then, cell pellets from two wells with the same transfection conditions were suspended in 400 μl of PBS and stored at −20°C until analysis.

Transfection efficiency was determined microscopically for each experiment by using 10 nM BLOCK-iT™ Alexa Fluor^®^ Red Fluorescent Control (Invitrogen).

### Enzyme-Linked Immunosorbent Assay for FANquantification

The concentration of FAN protein was determined using NSMAF Human ELISA Kit (Cloud-Clone Corp.) for human fibroblasts or ELISA kits (EIAab) for mouse fibroblasts, respectively, according to the manufacturer’s protocols. Absorbance measurements were performed on a BioTek SynergyHT plate reader with the wavelength set at 450 nm. The concentration of FAN in the tested samples was calculated based on the standard curve and expressed in ng/mL.

### Animals

Twenty DBA/1 male mice were used in the experiments, conducted according to Local Ethical Committee consent (Act No. WAW2/083/2019). Seven-week-old mice (Janvier Labs) after 1 week of adaptation, were randomly divided into four experimental groups. Experimental animals were subjected to both CIA induction and mu_miR172a administration (CIA + miRNA), *n* = 8. Control animals were 1) not treated (NT), *n* = 4; 2) subjected to mu_miR172a administration (miRNA), *n* = 4; or 3) subjected to CIA induction (CIA), *n* = 4.

### Experimental Design

**FIGURE 1:**
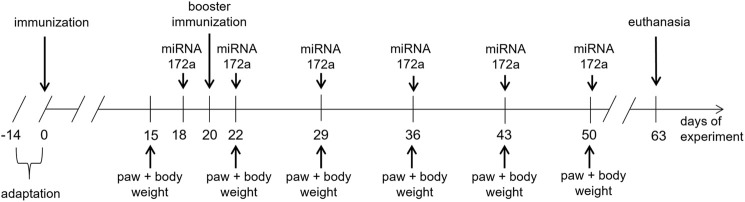
Design of the experiment with the use of an animal model of RA–CIA.

The Experimental Design is Presented in Figure 1.

### CIA Induction

In experimental animals, CIA was induced according to protocols (Brand et al., 2007; Bevaart et al., 2010b). On Day 0, animals were subjected to anesthesia (ketamine, 100 mg/kg, xylazine, 10 mg/kg, intraperitoneally). Next, mice were injected subcutaneously into the tail, 1 cm below the tail base, with 100 µl of immunization mixture (1 mg/ml bovine collagen II, Chondrex, in complete Freund’s adjuvant, 2 mg/ml, DIFCO).

On day 20 (3rd week), animals were anaesthetized as previously and injected subcutaneously, 0.5 cm below the previous injection, with100 µl of booster immunization mixture (1 mg/ml bovine collagen II in incomplete Freund’s adjuvant, Santa Cruz).

The immunization mixtures were prepared by intense mixing between two syringes connected with luer-luer connections of collagen II solution with complete or incomplete Freund’s adjuvant (1:1) until the consistency of thick cream was obtained.

### mu_miR172a Administration

Two days before the booster injection, experimental animals received a subcutaneous injection (into the neck area) of mu_miR172a (0.2 mg/ml in 0.9% NaCl). Injection was repeated 2 days after the booster injection and repeated once a week. Animals received a total of six doses of mu_miR172a.

### Paw Thickness Measurement

Beginning a week preceding booster injection (2nd week), once a week, paw thickness was measured. Joints of both left and right and front and hind paws were measured: metacarpus (height and width), metatarsus (height and width), and ankle joint (height and width). Means of 1) width and 2) height of both the left and right (L + R) metacarpus, metatarsus and ankle joint of animals from each experimental group in the following weeks were calculated. Finally, the ratio of width and height of both the left and right (L + R HxW) metacarpus, metatarsus and ankle joint of animals from each experimental group in the following weeks was calculated.

### Body Weight

Along with paw thickness measurements, the body weights of the mice were obtained. Means of the body mass of animals from each experimental group in the following weeks were quantified.

### Statistical Analysis

Measurements of paw thickness and body mass of mice, which are the indicators of the disease development and treatment efficiency, were subjected to statistical analysis. Ratios of width and height of both the left and right (L + R HxW) metacarpus, metatarsus and ankle joint of animals from each experimental group in the following weeks (see Materials and Methods section: Paw thickness measurement) were counted for comparison.

The mean and the standard deviation (SD) for variables (ratios and body mass) were reported in the following weeks of the experiment. The variables were compared with the two-way ANOVA test or mixed models (in case of missing values). The significance level was set at 0.05. The statistical significance is marked in the figure as follows: *p < 0.05; **<0.01; ***p < 0.001.

## Results

The bioinformatics analysis pointed on bol-miR172 as the miRNAs molecule from cabbage leafs having potential to regulate the expression of the FAN protein encoding gene. Molecules bol-miR172a and bol-miR172b of the same mature sequence AGA​AUC​UUG​AUG​AUG​CUG​CAU, present in a large quantities in cabbage leaves, have a sequence complementarity to human FAN mRNA binding site in the CDS region with a sequence complementarity of 89.4% were selected to interact with the mRNA encoding FAN protein. The calculated minimum free energy of the structure equals to −24.16 kcal/mol.

Here, we examined the effect of synthetic miR172 mimics on FAN protein expression in cell cultures and next examined their influence on ameliorating disease symptoms in an animal RA model, CIA.

### Human and Mouse Cell Lines

To determine the influence of plant miR172a on the regulation of mammalian FAN protein expression, we examined the effect of synthetic miR172 mimics on the level of FAN protein in selected human and mouse cell lines upon inflammation-inducing factor treatment.

We first examined the level of FAN protein in human foreskin fibroblast K21 cells after stimulation by LPS or TNF-a treatment. As shown in Supplementary Figure S1, the expression of FAN was significantly higher upon LPS treatment. Thus, subsequent experiments were conducted using LPS as an inducing agent. It was shown that the effect of miR172a addition was more pronounced in cells incubated for prolonged periods after transfection (24 vs. 72 h; Supplementary Figure S2). Additionally, single-stranded miR172a was almost ineffective in comparison with the same double-stranded molecule (Supplementary Figure S3).

**FIGURE 2:**
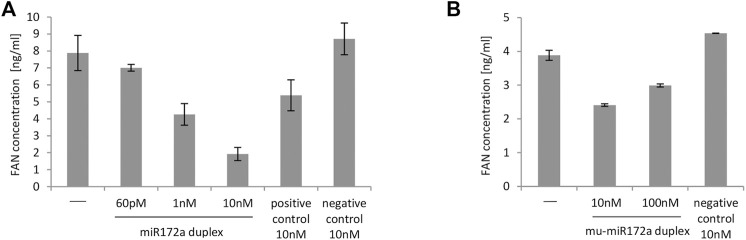
Level of FAN protein in K21 cells **(A)** and MEFs **(B)** transfected with miR172a duplex at different concentrations. **(A)** MISSION microRNA mimics and siRNA HMC0003 were used as negative and positive controls, respectively. In all cases, 4 h before the end of the experiment, LPS at a final concentration of 1 μg/ml was added to each well on plates. The first bar on the graph represents untransfected cells induced with LPS in the same way as cells transfected with the miR172a duplex or with controls. The FAN concentration was determined 48 h after transfection. **(B)** Nonspecific dsRNA (customized siGENOME Nontargeting siRNA #3, Dharmacon) was used as a negative control. In all cases, 4 h before the end of the experiment, 1 ng/ml TNF was added to induce the inflammatory process. The first bar on the graph represents cells not transfected with TNF in the same way as cells transfected with the mu_miR172a duplex and negative control. The FAN concentration was determined using dedicated ELISA Kits. The graphs show data obtained in single experiments with SD from ELISA measurements.

Figure 2A shows summarized data of FAN levels obtained after 48 h of incubation of K21 cells stimulated with LPS. A 10 nM concentration of duplex miR172a caused the greatest reduction in FAN levels when compared with negative or untreated controls. Notably, a concentration as low as 1 nM ds miR172a resulted in a decrease in FAN levels below the level acquired with positive control siRNA.

In further analyses, we tested the effect of the miR172 mimic, the customized mu_miR172a sequence, on mouse cells.

MEFs were transfected with different concentrations of mu_miR172a/mu_miR172a* duplexes (twice every 24 h) and then stimulated with TNF-α. As shown in Figure 2B, the presence of the mu_miR172a duplex markedly decreased the level of FAN protein in TNF-a-treated MEFs compared to cells not transfected with any miRNA or transfected with nonspecific dsRNA. The concentration of 10 nM mu_ miR172a was the most efficient among all tested and therefore became the basis for calculating the miRNA mimic dose administered to the experimental animals.

It should be mentioned however, that the above described experiments were performed repeatedly, and a similar dependency between the tested miR172 mimics and controls was observed. As experiments differed in the level of transfection efficiency and level of cell viability, we decided to present data from one representative experiment instead of combining data from a few experiments into one graph.

Having found that plant-derived miR172a mimics modulate the level of FAN protein in human and mouse cells, we undertook preliminary trials in an animal model of rheumatoid arthritis.

### Animal Studies

To evaluate the anti-inflammatory effect of mu_miR172a in animals, we administered mu_miR172a subcutaneously to mice with an evoked model of rheumatoid arthritis (CIA).

**FIGURE 3:**
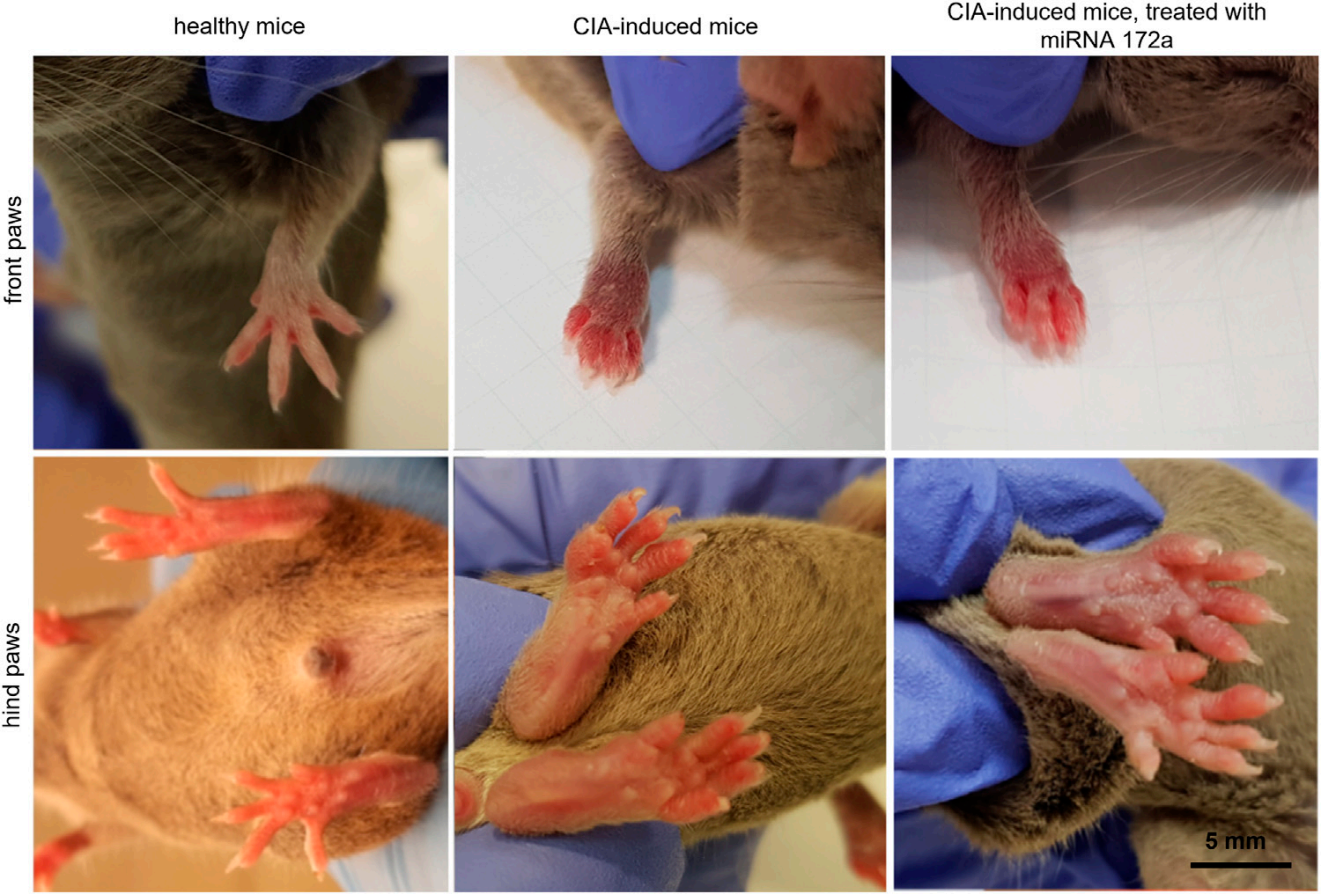
Paw oedema at the 4th week after immunization.

Beginning at the 2nd week after inducing CIA, body mass and paw thickness in mice were measured. Figure 3 presents the comparison of the metacarpus and metatarsus of control animals and metacarpal and metatarsal oedema observed in experimental (treated and untreated) mice.

### Paw Thickness and Body Mass

**FIGURE 4:**
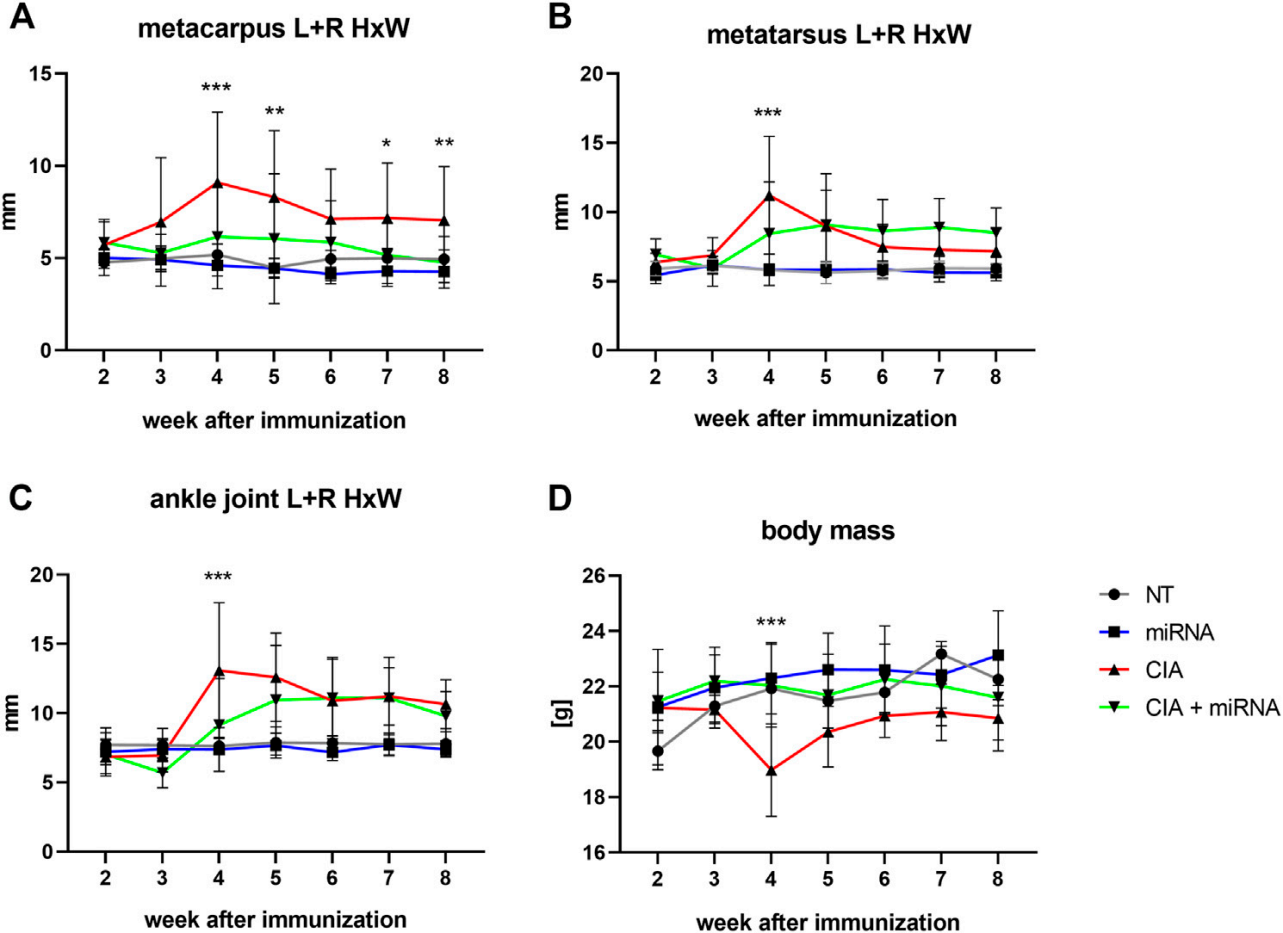
The ratio of width and height of the metacarpus **(A)**, metatarsus **(B)**, and ankle joint **(C)** and mean body mass **(D)** of experimental animals in the following weeks of the experiment. Statistical significance for CIA vs. CIA + miRNA marked on the graph. For statistical significance between other groups, see Supplementary Material (Supplementary Table S1). The statistical significance is marked in the figure as follows: *p < 0.05; **<0.01; ***p < 0.001. *n* = 4 (NT, miRNA, CIA), *n* = 8 (CIA + miRNA).

The metacarpus (A), metatarsus (B) and ankle joint (C) size and mean body mass (D) of control and experimental (treated and untreated) mice in following weeks were presented on the Figure 4.

There were no differences in the size (ratios of height and width) of the metacarpus, metatarsus or ankle joint (Figures 4A–C respectively) between healthy not treated (NT) animals and healthy animals treated with mu_miR172a (miRNA) during the experiment.

Beginning at week 3, the size of the metacarpus (Figure 4A) of CIA mice was significantly increased compared to that of healthy mice (NT, miRNA). In the following weeks, except for the 6th week, the size of the metacarpus of CIA mice was significantly increased compared to that of all other experimental groups (NT, miRNA, CIA + miRNA). There were no significant differences in the size of the metacarpus of CIA mice treated with mu_miR172a (CIA + miRNA) compared to healthy animals (NT, miRNA), except for the 6th week, where the size was increased significantly in comparison to healthy, mu_miR172-treated mice (miRNA).

At the 4th week, there was a significant increase in the size of the metatarsus (Figure 4B) of CIA mice, both not treated (CIA) and mu_miRNAa treated (CIA + miRNA), compared to healthy animals (NT, miRNA). This was observed for 2 weeks for CIA mice (CIA) and until the end of the experiment for CIA mice treated with mu_miR172 (CIA + miRNA). At the 4^th^ week, the size of the metatarsus of CIA mice (CIA) was significantly increased compared to that of CIA mice treated with mu_miR172a (CIA + miRNA).

Beginning at the 4th week, there was a significant increase in the size of the ankle joint (Figure 4C) of CIA mice (CIA) compared to all other experimental groups (NT, miRNA, CIA + miRNA). Beginning with a 5th week increase in the size of the ankle joint in CIA mu_miR172a-treated mice (CIA + miRNA) compared to healthy animals (NT, miRNA), no significant difference between nontreated (CIA) and mu_miR172a-treated (CIA + miRNA) mice was observed until the end of the experiment.

During the course of the experiment, the body mass (Figure 4D) of the healthy (NT, miRNA-treated) animals increased slightly similarly. The CIA animals with administered mu_miR172a (CIA + miRNA) showed no changes in body mass. A significant drop in body mass was observed in CIA animals (CIA) after the booster injection in the 3rd week compared to other experimental groups, remaining lower until the end of the experiment.

## Discussion

Since the first pioneering papers of Chen et al. (2008) and Zhang et al. (2011), there has been considerable discussion regarding whether the transfer of plant miRNAs from the diet to the blood truly occurs [reviewed in (Mar-Aguilar et al., 2020)]. The presence of plant miRNAs in the milk of breastfeeding human and animal mothers, proven by differing experimental setups, seems to constitute convincing evidence that this is indeed the case. The use of noncoding RNAs as therapeutics has recently been found to be an attractive alternative in the treatment of many diseases (Winkle et al., 2021). The question of whether miRNAs of plant origin constitute (or can be used as) “natural” drugs (Lukasik and Zielenkiewicz, 2016) remains unresolved.

In this paper, we have shown that, as identified by bioinformatics methods, miRNA172a from cabbage leaf juice (known in folk medicine as an anti-inflammatory agent) reduces the expression of the FAN protein in human cells, thus lowering the proinflammatory activity of TNF-α.

Based on the results obtained from the *in vitro* study, we decided to test the effect of miRNA172 in animals. Knowing that miRNA172 regulates the TNF-α signaling pathway *via* the FAN protein, we chose an animal model of rheumatoid arthritis, as the disease is strongly dependent on TNF-α signaling.

DBA/1 mice with CIA, an RA animal model, were injected with mu_miR172a subcutaneously. The results obtained from our study showed that mu_miRNA172a administration reversed or diminished the effect of CIA induction in experimental animals. The body mass of the CIA experimental animals subjected to mu_miRNA172a administration was reversed to the levels observed in the control animals, while CIA animals showed a decrease in body mass over the course of the disease. For paw thickness, the best results were observed in the front paws of mice, where metacarpal oedema was significantly reduced by mu_miRNA172a administration to CIA animals. Hind paw observations revealed that at least the peak oedema observed at the 4th week of the experiment after booster injection in CIA mice was strongly reduced, even with some oedema of the metacarpal and ankle joints observed in mu_miRNA172a-treated CIA mice.

These results support our hypothesis that administration of miR172a in patients with RA may be beneficial in diminishing the symptoms of the disease.

Published studies on the influence of miRNAs on RA or its animal models mostly analyze the levels of mammalian miRNAs. Additionally, different pathways engaged in the pathomechanism of RA were analyzed. Zhang et al. (2021) showed that miR-26b-5p influences CIA development and RA course by inhibiting Th17 cells, which are the main players in inflammatory responses. Similar properties of let-7g-5p were presented by Yang et al. (2020). miR-147 was shown to be engaged in inflammatory response induction, while its loss diminished joint inflammation in rats with pristine induced arthritis and reduced TNF-α, IL-6, MMP3, and MMP13 expression *in vitro* (Jiang et al., 2021). Another study showed that microRNA 23 is engaged in RA pathogenesis by influencing the NF-κB signaling pathway and decreasing TNF-α, IL-1β and IL-8 expression in RA patients (Gao et al., 2021).

To date, no data are available on the influence of plant-derived miRNAs on RA/RA animal models. However, there is rising evidence of the presence of plant (dietary) miRNAs in animal tissues (Liang et al., 2015; Yang et al., 2015; Lukasik and Zielenkiewicz, 2016; Benmoussa and Provost, 2019). Cabbage miR172 was found in mouse; after passage through the gastrointestinal tract, miR172 was detected in the blood, spleen, kidney and liver of the animals (Liang et al., 2014). Based on *in silico* analysis, plant miRNAs may interfere with human mRNA, thus regulating gene transcription at the posttranscriptional level and serving as therapeutic agents (Lukasik and Zielenkiewicz, 2016; Sanchita et al., 2018; Zhao et al., 2018). This supports the hypothesis of the possible use of plant miRNAs as therapeutic agents in human diseases.

## Data Availability

Publicly available datasets were analyzed in this study. This data can be found here: www.ebi.ac.uk.
