# Supplementary material for: The Anti-Inflammatory Effect of Cabbage Leaves Explained by the Influence of bol-miRNA172a on FAN Expression
Source: Front Pharmacol. 2022 Mar 24;13:846830. doi: 10.3389/fphar.2022.846830 (PMC8987499; doi:10.3389/fphar.2022.846830)
Supplement: Supplementary file 1 [file DataSheet1.PDF]

Supplementary Material

1 Supplementary Materials and Methods

1.1 miRNAs

The miR172a and miR172a* sequences were synthesized on the basis of bol-miR172a from *Brassica oleracea* var. c*apitata*:

miR172a: 5'-AGAAUCUUGAUGAUGCUGCAU-3'

The mu_miR172a and mu_miR172a* sequences were designed by changing 3 nt (underlined) inside the region corresponding to the mouse FAN mRNA sequence potential binding site to preserve complementarity:

mu_miR172a: 5'-AGAAUCUUAAGAAUGCUGCAU-3'

The miR172 mimics were chemically synthesized in the form of miR172a/miR172a* and mu_miR172a/mu_miR172a* duplexes with both 5' RNA strands phosphorylated and 3' ends protected by 2'-O-methyl uridine or 2'-O-methyl adenosine, respectively, and HPLC purified (FutureSynthesis, Poland). Alternatively, single-stranded miR172 mimics were synthesized and protected similar to the description above.

The following synthetic miRNA mimics were used in this study as controls:

siGENOME Non-Targeting siRNA #3, Dharmacon

MISSION microRNA Mimics HMC0003, Sigma

Hs_NSMAF_3, QIAGEN

1.2 Cell line cultures

Human foreskin fibroblasts (K21) were cultured in MEM/EBSS medium (HyClone) supplemented with nonessential amino acids (Gibco) and 10% inactivated FBS (Lonza) at 37 °C in a 5% CO_2 atmosphere.

Mouse embryonic fibroblasts (MEFs) were propagated using standard cell culture techniques and maintained in DMEM (Gibco) supplemented with 10% FBS (Gibco), 100 U/ml penicillin and 100 µg/ml streptomycin (Gibco) at 37 °C in a humidified atmosphere with 5% CO_2.

1.3 K21 transfection

One day before transfection, fibroblasts were plated on 6-well plates (2.8 x 10^5 cells/well). The next day, the medium was exchanged for fresh medium (1.75 ml). For each transfection sample, the miR172a-lipofectamine RNAiMAX (Invitrogen) complex was prepared as follows: the miR172a/miR172a* duplex was diluted in 125 µl of medium without serum (final concentration of miRNA when added to cells was 60 pM, 1 nM or 10 nM) and mixed gently with lipofectamine RNAiMAX (7.5 µl) diluted in 117.5 µl of medium without serum. After 5 min of incubation at room temperature, the miR172a-lipofectamine RNAiMAX complex was added to the cells. As a negative control, MISSION® microRNA Mimics (HMC0003, Sigma) was used at a final concentration of 10 nM, and untreated cells (without transfection) were used. A positive control siRNA designed and synthesized based on the NSMAF gene sequence Hs_NSMAF_3 (QIAGEN) was used. The cells were incubated for 48 or 72 hours at 37 °C in a 5% CO_2 atmosphere. Transfection efficiency was determined microscopically for each experiment by using 10 nM BLOCK-iT™ Alexa Fluor® Red Fluorescent Control (Invitrogen). Four hours before the end of the experiment, LPS (Sigma) at a final concentration of 1 µg/ml was added to each well of the plate and to untransfected cells, which were used as a control. After 4 hours, the cells were detached using trypsin and washed 3 times in PBS. After centrifugation, the cell pellet was suspended in 400 µl of PBS and stored at -20 °C until analysis.

1.4 Transfection of MEFs

MEFs were plated on 24-well plates (1,9x10^4 cells/well) one day before transfection. The next day, 1 nM, 10 nM or 100 nM mu_miR172a/mu_miR172a* duplex (heated at 80 °C for 3 minutes and then cooled down at RT) was transfected into MEFs using ScreenFect A-plus (ScreenFect GmbH) according to the manufacturer's instructions. Each concentration of miRNAs was used to transfect two wells. Four to five hours later, the medium was discarded and replaced with complete fresh medium. The next day, the transfection procedure was repeated, and the cells were incubated for the next 24 h. As a negative control, nonspecific dsRNA (customized siGENOME Nontargeting siRNA #3, Dharmacon) at a concentration of 10 nM was used. Transfection efficiency was determined microscopically for each experiment by using 10 nM BLOCK-iT™ Alexa Fluor® Red Fluorescent Control (Invitrogen). Four hours before the end of the experiment, TNF-α (Sigma) at a concentration of 1 ng/ml was added to each well, and untransfected cells were used as a control. After 4 h of incubation with TNF, cells were detached with trypsin (Sigma), centrifuged and washed 3 times in PBS (Invitrogen). Pellets from 2 wells with the same transfection conditions were suspended in 400 µl of PBS and stored at -20 °C for further analysis.

2 Supplementary Figures and Tables

2.1 Supplementary Figures

Supplementary Figure 1. Level of FAN protein in K21 cells after stimulation by LPS or TNF-α.

The first bar on the graph represents cells without any stimulation. FAN concentration was determined 72h after seeding cells on plate using NSMAF Human ELISA Kit (Cloud-Clone Corp.). Absorbance was measured at 450 nm. Graph shows data obtained in one experiment with SD from ELISA.

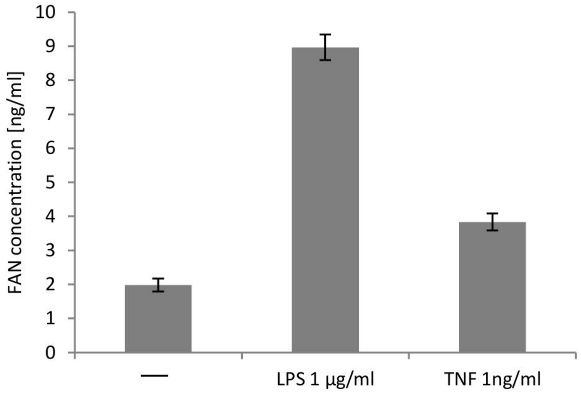

Supplementary Figure 2. Level of FAN protein in K21 cells transfected with miR172a duplex in concentration 60pM or 1nM.

In all cases, 4 hours before the end of experiment LPS (Sigma) at final concentration 1 µg/ml was added to each well on plate. The first bar on the graph represents not transfected cells induced with LPS in the same way as cells transfected with miR172a duplex. FAN concentration was determined 24h and 72h after transfection using NSMAF Human ELISA Kit (Cloud-Clone Corp.) and absorbance was measured at 450 nm. Graph shows data obtained in one experiment with SD from ELISA.

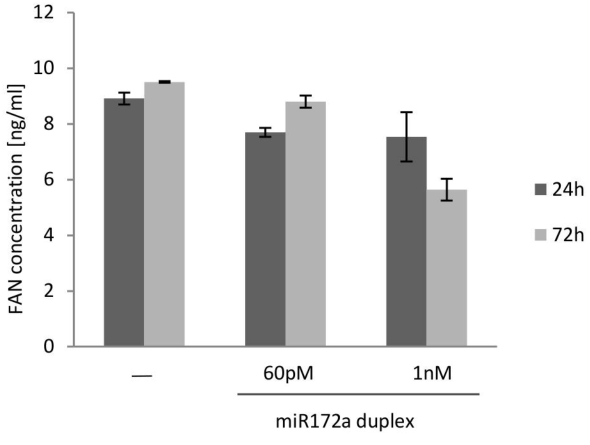

Supplementary Figure 3. Level of FAN protein in K21 cells transfected with miR172a duplex or single-stranded miR172a.

MISSION microRNA Mimics (HMC0003, Sigma) in final concentration 10nM was used as negative control. In all cases, 4 hours before the end of experiment LPS (Sigma) at final concentration 1 µg/ml was added to each well on plate. The first bar on the graph represents not transfected cells induced with LPS in the same way as cells transfected with miR172 or with negative control. FAN concentration was determined 48 h after transfection using NSMAF Human ELISA Kit (Cloud-Clone Corp.) and absorbance was measured at 450 nm. Graph shows data obtained in one experiment with SD from ELISA.

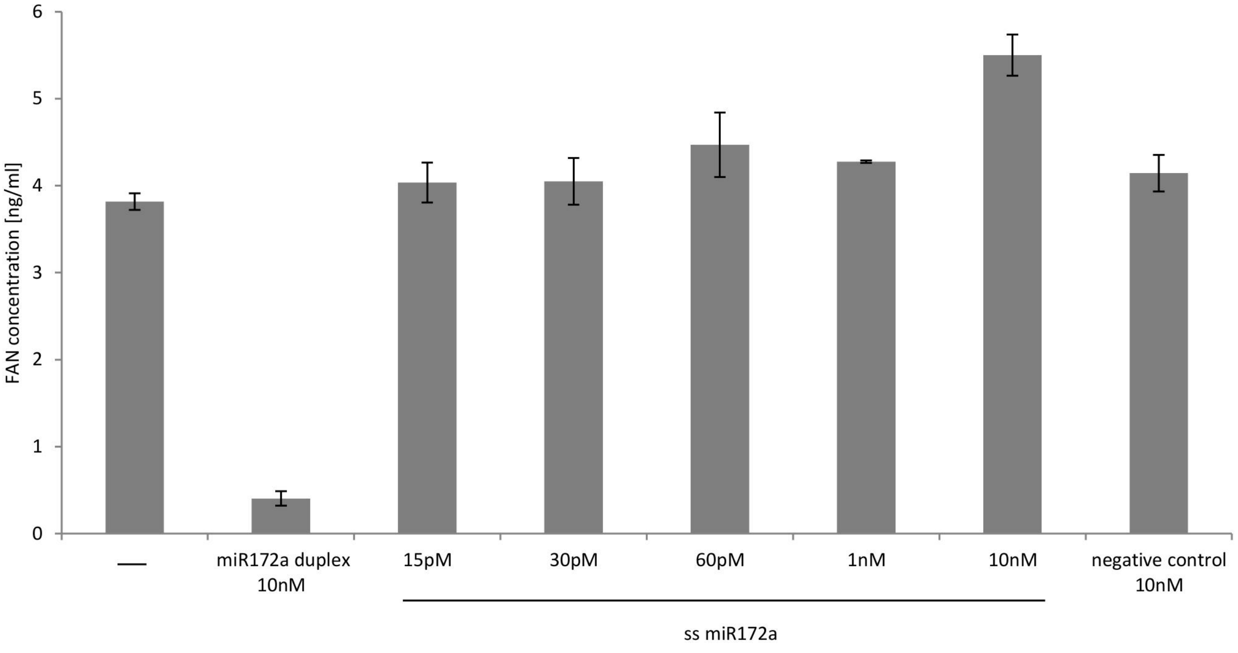

2.2 Supplementary Tables

Supplementary Table 1. Statistical significance between groups of experimental animals for body mass, metacarpus L+R HxW, metatarsus L+R HxW and ankle joint L+R HxW in following weeks of experiments. The statistical significance marked as follows: *p<0.05; **<0.01; ***p< 0.001. n=4 (NT, miRNA, CIA), n=8 (CIA + miRNA).

	**Statistical significance**			
	Body mass	metacarpus L+R HxW	metatarsus L+R HxW	ankle joint L+R HxW
**2 week**				
**NT vs. miRNA**	ns	ns	ns	ns
**NT vs. RA**	ns	ns	ns	ns
**NT vs. RA + miRNA**	*	ns	ns	ns
**miRNA vs. RA**	ns	ns	ns	ns
**miRNA vs. RA + miRNA**	ns	ns	ns	ns
**RA vs. RA + miRNA**	ns	ns	ns	ns
**3 week**				
**NT vs. miRNA**	ns	ns	ns	ns
**NT vs. RA**	ns	*	ns	ns
**NT vs. RA + miRNA**	ns	ns	ns	*
**miRNA vs. RA**	ns	*	ns	ns
**miRNA vs. RA + miRNA**	ns	ns	ns	ns
**RA vs. RA + miRNA**	ns	ns	ns	ns
**4 week**				
**NT vs. miRNA**	ns	ns	ns	ns
**NT vs. RA**	**	***	***	***
**NT vs. RA + miRNA**	ns	ns	**	ns
**miRNA vs. RA**	***	***	***	***
**miRNA vs. RA + miRNA**	ns	ns	**	ns
**RA vs. RA + miRNA**	***	***	***	***
**5 week**				
**NT vs. miRNA**	ns	ns	ns	ns
**NT vs. RA**	ns	***	***	***
**NT vs. RA + miRNA**	ns	ns	***	**
**miRNA vs. RA**	*	***	**	***
**miRNA vs. RA + miRNA**	ns	ns	***	***
**RA vs. RA + miRNA**	ns	**	ns	ns
**6 week**				
**NT vs. miRNA**	ns	ns	ns	ns
**NT vs. RA**	ns	*	ns	**
**NT vs. RA + miRNA**	ns	ns	***	***

**miRNA vs. RA**	ns	**	ns	***
**miRNA vs. RA + miRNA**	ns	*	***	***
**RA vs. RA + miRNA**	ns	ns	ns	ns
**7 week**				
**NT vs. miRNA**	ns	ns	ns	ns
**NT vs. RA**	ns	*	ns	**
**NT vs. RA + miRNA**	ns	ns	***	***
**miRNA vs. RA**	ns	**	ns	**
**miRNA vs. RA + miRNA**	ns	ns	***	***
**RA vs. RA + miRNA**	ns	*	ns	ns
**8 week**				
**NT vs. miRNA**	ns	ns	ns	ns
**NT vs. RA**	ns	*	ns	*
**NT vs. RA + miRNA**	ns	ns	**	*
**miRNA vs. RA**	*	**	ns	**
**miRNA vs. RA + miRNA**	*	ns	***	*
**RA vs. RA + miRNA**	ns	**	ns	ns

Supplementary Table 2. Content of miR172 in different cabbage RNA sources.

Micro RNA was isolated with miRVana isolation kit (InVitrogen) performed according to the manufacturer protocol. Concentration of miR172 was estimated by quantitative PCR analysis (qRT-PCR reactions) performed with TaqMan ® Universal PCR Master Mix (no AmpErase ® UNG kit; Applied Biosystems) as described in Lukasik et al. Detection of Plant miRNAs Abundance in Human Breast Milk. Int. J. Mol. Sci. 2018, 19, 37; doi:10.3390/ijms19010037

Source (equivalent of 100 mg)	miRNA concentration (ng/µl)	miR172 concentration (pM)
Whole fresh leaves	82,0 – 134,8	87,0 – 225,4
Juice from fresh leaves	38,6 – 76,1	2,6 – 21,53
Juice from sauerkraut	76,3 – 98,2	0,04 – 0,22
Commercial extract(s)	64,0 – 298,0	0,14 – 1,03
